# Patient-reported experience of clinical care of osteogenesis imperfecta (OI) during the COVID-19 pandemic

**DOI:** 10.3389/fpubh.2022.951569

**Published:** 2023-01-04

**Authors:** Debra Smyth, Monica Hytiris, Coreen Kelday, Ciara McDonnell, Christine Burren, Adrian Gardner, Lisa Mills, Susan Parekh, Oliver Semler, Angela Stewart, Ingunn Westerheim, Muhammad Kassim Javaid, Patricia Osborne, S. Faisal Ahmed

**Affiliations:** ^1^School of Medicine, Dentistry and Nursing, Office for Rare Conditions, University of Glasgow, Glasgow, United Kingdom; ^2^Brittle Bone Society, Dundee, United Kingdom; ^3^Department of Pediatric Endocrinology and Diabetes, Children's Health Ireland, Dublin, Ireland; ^4^Department of Pediatric Endocrinology and Diabetes, Bristol Royal Hospital for Children, University Hospitals Bristol and Weston NHS Foundation Trust, Bristol, United Kingdom; ^5^Orthopedic Department, Royal Orthopedic Hospital, NHS Foundation Trust, Birmingham, United Kingdom; ^6^Pediatric Department, Bristol Royal Hospital for Children, University Hospitals Bristol and Weston NHS Foundation Trust, Bristol, United Kingdom; ^7^Department of Pediatric Dentistry, UCL Eastman Dental Institute, University College London, London, United Kingdom; ^8^Department of Pediatrics, University Hospital Cologne, University of Cologne, Cologne, Germany; ^9^Osteogenesis Imperfecta Federation Europe, Oslo, Norway; ^10^Nuffield Department of Orthopedics, Rheumatology and Musculoskeletal Sciences, Nuffield Orthopedic Center, University of Oxford, Oxford, United Kingdom

**Keywords:** COVID-19, osteogenesis imperfecta (OI), pandemic, rare conditions, rare diseases, remote consultation

## Abstract

**Background:**

Research on the effects of the COVID-19 pandemic on people with rare diseases is limited. Few studies compare healthcare throughout the progression of the ongoing pandemic.

**Aims:**

To assess the impact of the pandemic on individuals with osteogenesis imperfecta across two consecutive years, understand what challenges were encountered, and analyse the experience of remote consultation.

**Methods:**

An initial survey was distributed following the first lockdown in August 2020, and a second survey in April 2021. The surveys explored four themes- effects on therapy, alternatives to consultation, effect on mental health, and perceived risks of COVID-19.

**Results:**

In the 2020 survey, of the 110 respondents, 69 (63%) had at least one appointment delayed due to the lockdown, compared with 89 of the 124 respondents (72%) in 2021. Of the 110 respondents in 2020, 57 (52%) had a remote consultation, increasing to 92 of 124 (74%) in the follow-up survey. In the 2020 survey 63 of 91 respondents (69%) expressed anxiety due to lockdown, compared with 76 of 124 (61%) in 2021. The percentage of total respondents expressing a preference for remote consultation was 48% in 2020, increasing to 71% in 2021.

**Conclusions:**

The pandemic has had widespread effects on the mental and physical health of those with OI. These effects, alongside appointment delays, have increased as the pandemic progresses. Encouragingly, the increasing preference for remote consultation may indicate that this could be a viable long-lasting alternative to face-to-face appointments, especially for patients who previously traveled vast distances for specialist care.

## Introduction

Although the direct effect of COVID-19 on those infected with the virus has been dramatic, the indirect effects of the pandemic on the delivery of healthcare have also been substantial and there is a need to investigate this further (1, 2). One group in particular who may have been affected are those with rare conditions who already felt under-supported and under-resourced (3) prior to this international health crisis. Rare conditions or rare diseases are defined in Europe as those conditions that affect less than 1 in 2000 people (4), with the majority being chronic, debilitating or even life-threatening (4). From an organizational point of view, lockdown pressures during the recent pandemic have resulted in delays in treatment and difficulties in accessing treatment (3, 5, 6), and additional pressures have been exerted on charities and support groups who frequently provide information and support (7). With new rules on public gatherings and social distancing, fundraising events have been canceled resulting in financial hardships for charities (1).

Osteogenesis imperfecta (OI) is a rare collagen disorder, which has a genetic origin and often presents in childhood with skeletal fragility. With a prevalence of about 1 in 15,000, it is expected that there will be around 5,000 individuals with a variable severity of OI in the UK (8, 9). Like many rare conditions, there is no cure for OI, however symptomatic treatments exist with the aim of optimizing mobility, strengthening bones and muscles, and reducing pain and the risk of fractures (9, 10). Research on the effects of the COVID-19 pandemic on the health care of people with rare diseases, including OI, is limited (1, 2), and to improve the understanding of these effects, the Brittle Bone Society (BBS) of the UK and the Republic of Ireland, a national charity that supports individuals and families affected by OI, performed surveys over two time periods in 2020 and 2021.

The aim of these surveys was to explore the impacts of the global COVID-19 pandemic on the osteogenesis imperfecta community, to understand the challenges they encountered throughout the ongoing pandemic, and to analyze the experience of remote consultations on clinical care.

## Methods

To measure the effects of the COVID-19 pandemic on patients with osteogenesis imperfecta, the BBS developed two online questionnaire surveys that were distributed from August to December 2020 and from March to April 2021. These questionnaires were circulated through its membership *via* social media (including Facebook, Twitter, and Instagram), the monthly BBS newsletters and posted on its website. The second survey was also circulated *via* OIFE (Osteogenesis Imperfecta Federation Europe) through their membership and online. The aim of these surveys was to assess the effect of the pandemic on healthcare received by patients with OI, but also to compare the effects of the pandemic between both periods. The questions were developed in conjunction with a working group of five individuals with varying types of OI, and with input from the BBS Medical Advisory Board, and looked at the following themes: theme one–effect on therapy; theme two–alternatives to face-to-face consultation; theme three–effect on mental health and well-being. The second survey also incorporated a novel theme; theme four which focused on the perceived risks of COVID-19. This was included only in the latter study due to a lack of knowledge of the effects of COVID-19 in OI in the first wave of the pandemic when the first survey was distributed, and the unavailability of vaccines at this point. In the primary survey, themes were identified from answers, and these themes became the basis for the questions in the second survey. Some questions in the second survey were also based on the common enquiries the BBS was receiving over the first year of the pandemic.

All members of the BBS (which covers the UK and Ireland) were invited to participate in the survey. There were no exclusion criteria for participating and respondents were encouraged to participate regardless of health experience during the pandemic. Open questions were used to analyse pandemic care, and responses included positive, negative and no impact options to prevent response bias. The survey was distributed *via* the application “Jotform” (Jotform Inc, CA, USA) to the 600 active members of BBS as well as its wider dissemination list of 1200 individuals. In addition, the survey was also distributed to the 5,600 followers of BBS on social media, and people from other countries were invited through the OI Federation Europe (OIFE) network. Each response was provided with an individual submission ID to prevent duplication of results and to ensure every response was unique. The questionnaire did not collect any identifiable information from respondents. The data from respondents were supplied to the investigators on an anonymous basis for retrospective analysis. Both questionnaires contained both quantitative and qualitative aspects, with multiple-choice and open-ended questions. The quantitative data were analyzed using descriptive statistics utilizing Microsoft Excel. The qualitative data were grouped using inductive thematic analysis (11). All respondents were included in the analysis, including those from outside the UK and Ireland.

## Results

### Demographics

The initial survey completed in August 2020 had 110 respondents. The follow-up survey completed in April 2021 had 124 respondents. Details of the contents of the surveys are included in Table 1. All respondents in the initial survey originated from the UK and Ireland with 85 (77%) living in England. Of these, 80 (73%) were individuals with OI and the remainder were a combination of parents, carers, and other relatives. The follow-up survey completed in April 2021 had 124 respondents, 96 (74%) from the UK and Ireland, with the majority, 79 (64%), from England. The remainder lived in Europe, South Africa, India, and the USA. Of these, 104 (84%) respondents were individuals with OI; 15 (12%) were parents of a child with OI, and 5 (4%) were carers (Table 2).

**Table 1:** Survey questions in August 2020 and April 2021.

**August 2020 survey questions**	**April 2021 survey questions**
**Demographics**	
Are you completing the survey as an individual with OI/A parent of someone with OI?	Are you completing the survey as an individual with OI/A parent of someone with OI?
Do you live in England/Northern Ireland/Wales/Scotland?	What type of OI do you/they have?
	Age of individual with OI
	Gender of individual with OI
	Country of residence
	Who do you /the individual regularly see for your OI (tick all that apply)
**Theme 1: Effects on therapy**	
Have you had any OI appointments postponed due to COVID-19?	Have you/the individual with OI ever had a surgical procedure postponed or canceled due to COVID-19 pandemic by the Hospital?
If yes, what was it for?	If you have had a surgical procedures postponed on average how long have these been postponed by in months?
How has this impacted you?	I/the individual with OI have had a scan, such as an x-ray, DXA, MRI delayed or canceled due to the COVID-19 pandemic by the hospital
Has COVID-19 impacted your transition to adult services?	I/the individual with OI have had a day unit treatment, such as IV infusion delayed or canceled due to the COVID-19 pandemic by the hospital
Has COVID-19 impacted your ability to get a wheelchair assessment?	I/the individual with OI have had an appointment with an allied healthcare professional (e.g., physio, OT, nurse, dental, hearing test) delayed or canceled due to the COVID-19 pandemic by the hospital
Has COVID-19 impacted your rehab? i.e., post-surgery/fracture.?	I/the individual with OI have had an appointment with a Consultant/Specialist delayed or canceled due to the COVID-19 pandemic by the hospital
Has COVID-19 impacted how you liaise with schools/further education?	If you have had any appointment postponed on average how long have these postponed by in months?
	If you receive support/care or personal assistance at home on a regular basis to what extent has this been impacted by the pandemic?
**Theme 2: Alternatives to consultation**	
Have you received remote consultations during lockdown?	Have you/the individual with OI received remote consultations/appointments during the COVID-19 pandemic?
Was the remote consultation *via*: Telephone/ Video/ Telephone and video:	Was the remote consultation/appointment *via*:
If Yes, was this your first remote consultation?	Going forward is this something you would like to see more of?
If Yes, how did you find this?	I/the individual with OI prefer a hospital appointment *via* telephone/video call to a face-to-face visit
Going forward, is this something you would like to see more of?	
Would you like to suggest any ways this experience could have been improved or do you have further comments?	
**Theme 3: Effect on mental health**	
How has COVID-19 affected you?	Please indicate on a scale of 1 to 5 how much you have struggled/or not struggled with the following
	> Weight and diet
	> Mobility
	> Pain
	> Anxiety/mental health
	> Keeping physically active
	> Not seeing friends and family
Please explain the impact COVID-19 has had on your mental health	
**Theme 4: Perceived risk of COVID-19 (only addressed in April 2021 survey)**	
In general would you say you/the person with OI's health is: Poor/Fair/Good/Very good/Excellent	
Have you/ the individual with OI received a letter from the NHS saying you have been identified as someone at risk of severe illness if you catch COVID 19 because of underlying health condition?	
If you answered no to this is it because: You have not been identified as at risk i.e. you have mild OI You are unsure if you should be in the at risk group but you did not feel the need to register You are unsure if you should be in the at risk group but you did not have access to healthcare Other	
Do you have any underlying health condition that would also identify you as being clinically extremely vulnerable if you caught COVID-19?	
Have you/ the individual been admitted to hospital due to having COVID-19?	
In the event that you have either have had COVID-19 or may catch COVID-19, in your opinion do you think you would be clinically more difficult to treat than someone that does not have any underlying health condition?	
In the event that you have either had COVID-19 or may catch COVID-19 - in your opinion do you think it did/would take you: Longer than someone without an underlying health condition Same length of time to recover as someone without an underlying health condition Unsure	
Have you/the individual with OI ever canceled or postponed an appointment or treatment due to COVID-19?	
Have you/the individual with OI ever had to switch or change any aspect of your current therapies to avoid hospital attendance due to COVID-19?	
Have you/the individual with OI ever decided not to go to Accident and Emergency with a suspected fracture to confirm diagnosis during the COVID-19 pandemic?	
To what extent has the pandemic affected you/the family's ability to leave the house?	
Have you/ the individual with OI had a COVID-19 vaccine?	
If you/the individual with OI has not had the vaccine why is this?	

**Table 2:** Patient demographics.

**2020 Survey (*n* = 110)**	***N***	**2021 Survey (*n* = 124)**	***N***
**Individual/parent/carer of someone with OI**			
Individual with OI	80	Individual with OI	104
Parent of someone with OI	15	Parent of someone with OI	15
Parent of someone with OI and individual with OI	12	Carer of someone with OI	5
Parent and carer of someone with OI	2		
Other	1		
**Country of residence**			
England	85	England	79
Scotland	11	Other	28*
Northern Ireland	6	Scotland	10
Wales	4	Ireland	4
Ireland	2	Northern Ireland	2
No response	2	Wales	1
**Severity of OI**			
Not asked in this survey		Mild	36
		Moderate	47
		Severe	36
		Unsure	5
**Age of individual**			
Not asked in this survey		Ages <15	11
		Ages 18–30	12
		Ages 31–50	54
		Ages 50–64	39
		>Ages 64	8

^*^Including Norway, Sweden, Denmark, Netherlands, Malta, Finland, India, USA, Belgium, South Africa.

### Theme 1: Effects on therapy

Of the 110 responses to the original 2020 survey, 69 (63%) participants had appointments postponed due to the lockdown, and of these 19 (28%) had more than one appointment postponed. Fifty-two reported a delay in a consultation, 22 had treatments deferred, and six had a surgery postponed. Of the 62 respondents who gave specific feedback on how these postponements had impacted their care, the five identified themes were: delays in treatments/ investigations (21 responses, 34%), adverse effects on health or increased pain (20 responses, 32%), anxiety and frustration (10 responses, 16%), and suitable arrangements were made or caused a minimal effect on care (11 responses, 18%). Of the 22 pediatric patients in this cohort, five (23%) had their transition to adult services impacted. Ten of the 38 respondents (26%) requiring a wheelchair assessment were unable to have this due to the lockdown. Twenty-eight of the 54 respondents (52%) who required rehabilitation (i.e., post-surgery or following a fracture) were unable to have this, with 15 of these mentioning lack of in-person physiotherapy being their main concern. Of the 39 respondents who had educational requirements during COVID-19, 23 (59%) felt the lockdown affected their links with educational services.

Of the 124 respondents in the follow-up 2021 survey, 89 (72%) had appointments postponed due to the lockdown, of these 36 (40%) had more than one appointment postponed. Twenty-two respondents (18%) had a surgical procedure postponed or canceled, 30 respondents (24%) had imaging delayed/canceled, 19 (15%) had day unit treatment postponed/canceled, 63 (51%) had an appointment with an allied healthcare professional canceled/postponed, and 64 (52%) had an appointment with a specialist delayed or canceled. Twenty-five respondents had not been given a new date for their medical appointment at the time of the survey (32%). For the postponed surgical procedures 12 (48%) respondents with delayed appointments have not been assigned a new date for their postponed procedure at the time of the survey. Of the 124 participants, 29 (23%) required regular support or personal assistance at home. Four of these (14%) had their hours of care completely cut, four (14%) had the majority of their hours cut, six (21%) had some of their hours cut, and the remainder (15, 52%) experienced no change in their hours of support.

### Theme 2: Alternatives to consultation

**Figure 1:**
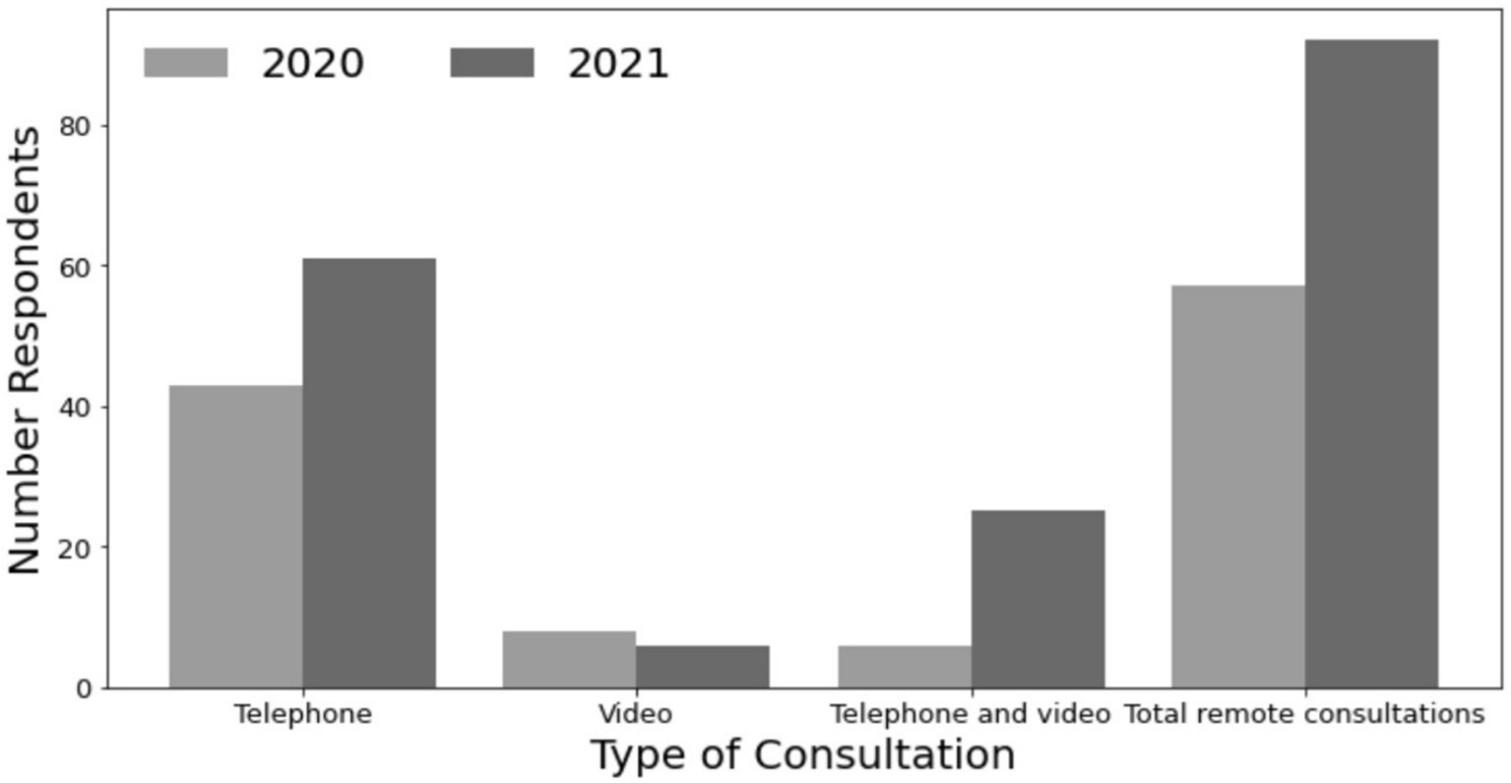
Type of consultation from 2020 to 2021. Comparison of consultation types from 2020 to 2021 survey. Overall, there were 57 remote consultations amongst 110 respondents in 2020 and 92 amongst 124 respondents in 2021. In the primary survey, 43 respondents had telephone consultations, compared with 61 in the 2021 survey. Eight respondents had a video consultation in 2020 and a further six in 2021. Six participants had a combination of video and telephone consultations in 2020 and a further 25 in the 2021 survey.

Of the 110 respondents in the 2020 survey, 57 (52%) received remote consultations during the lockdown. For 51 (89%) respondents this was their first experience of remote consultation. The majority of these were telephone consultations (43 responses, 75%), eight (14%) were *via* video consultation, and the remaining six (11%) were a combination of both. Experience with remote consultations was rated as excellent by 15 (26%), very good by 13 (23%), good by 17 (30%), not good by 7 (12%) and poor by 5 respondents (9%). When asked about future preferences, 43 out of the 90 (48%) respondents to this section answered they would like to have a remote consultation in the future. Of the 57 who had a remote consultation, areas for improvement that were reported included the choice of type of consultation by nine (27%), lack of examinations and blood tests by six (18%) and not being given a time for the phone call by four (12%). Of the 124 respondents in the 2021 survey, 92 (74%) respondents had received a remote consultation/appointment. Of the 92 remote consultations, telephone consultations were performed in 61 (66%), video in six (7%), and a combination in 25 (27%). Types of consultations compared across both surveys can be seen in Figure 1. When the 124 respondents were asked about future preferences, in total, 87 (71%) said they would like to see more remote consultations with 64 of these 87 (74%) stating they would prefer remote consultations but not for every appointment, compared with 48% in 2020.

### Theme 3: Effect on mental health

In the 2020 survey, respondents were asked how COVID-19 affected them. The following themes were identified: shielding and isolation (*n* = 24); anxiety about leaving the house (*n* = 18); inability to obtain equipment/treatment (*n* = 12); reluctance to seek medical attention/ longer waits (*n* = 9); reduction inability to exercise (*n* = 8); and financial hardship (*n* = 2). When asked specifically how the lockdown had affected their mental health, of the 91 respondents to this section, 63 (69%) expressed increased anxiety and depression, 12 (13%) expressed concerns about not seeing friends and family, and two (2%) struggled with not being physically active. Seven (8%) respondents reported no change in their mental health due to the lockdown, and six (7%) reported a positive change in their mental health. In the 2021 survey when asked how the lockdown affected participants, of the 124 respondents, 55 (44%) struggled moderately to significantly with weight and diet; 70 (56%) struggled moderately to significantly with mobility; 76 (61%) struggled moderately to significantly with anxiety/mental health; 103 (83%) struggled moderately to significantly with not seeing friends and family; 71 (57%) struggled moderately to significantly with pain; 89 (72%) struggled moderately to significantly with keeping physically active. For the 29 participants with carers, 22 (76%) struggled moderately to significantly with anxiety and mental health, when compared to those who did not have carers, 54 (44%) struggled moderately to significantly with anxiety/mental health.

### Theme 4: Perceived risks of COVID-19

This theme was only explored in the 2021 survey. When asked about their general state of health, of 124 participants, 37 (30%) felt their general health was excellent or very good, 50 (40%) felt their health was good, 26 (21%) felt their health was fair and 11 (9%) of respondents felt their overall health was poor. Thirty-four respondents (27%) had received an official letter from their healthcare provider advising them they were at risk of severe COVID-19 related illness because of an underlying health condition, whilst 69 (56%) self-identified as having an underlying health condition that would make them clinically vulnerable if they caught COVID-19. Of the 63 who did not receive a letter, the reasons were: 25 (40%) had not been identified as at risk (i.e., had mild OI); 13 (21%) were unsure if they were in the at-risk group but did not feel the need to register; nine (14%) were unsure if they should be in the at risk group but did not have access to healthcare. Three respondents of 121 respondents to this question (2%) were admitted to hospital due to having contracted COVID-19. Of the 117 who responded to the question regarding contracting COVID-19, 81 (69%) participants felt they would be clinically more difficult to treat than someone who does not have an underlying health condition, whilst 25 (21%) were unsure. Of the 120 who were asked about recovery from COVID-19, 77 (64%) felt it would take them longer than someone without an underlying health condition to recover, whilst 24 (20%) were unsure. When asked for further comments on recovery, 27 participants responded in total. Six respondents mentioned fear of rib fractures, from coughing or in hospital care, three mentioned that healthcare professionals have said their risk of death is high, and two mentioned fear of not being accepted into ICU or not being prioritized for care due to their disability. Due to perceived fear of COVID-19, of the 124 participants, 58 (47%) postponed their own appointments, 54 (44%) said they had switched or changed aspects of their current therapies to avoid hospital attendance, 25 (20%) reported that they decided not to seek out of hours emergency input at the hospital for a suspected fracture and 66 (53%) had not left their home at all, or for most of the time since March 2020.

**Figure 2:**
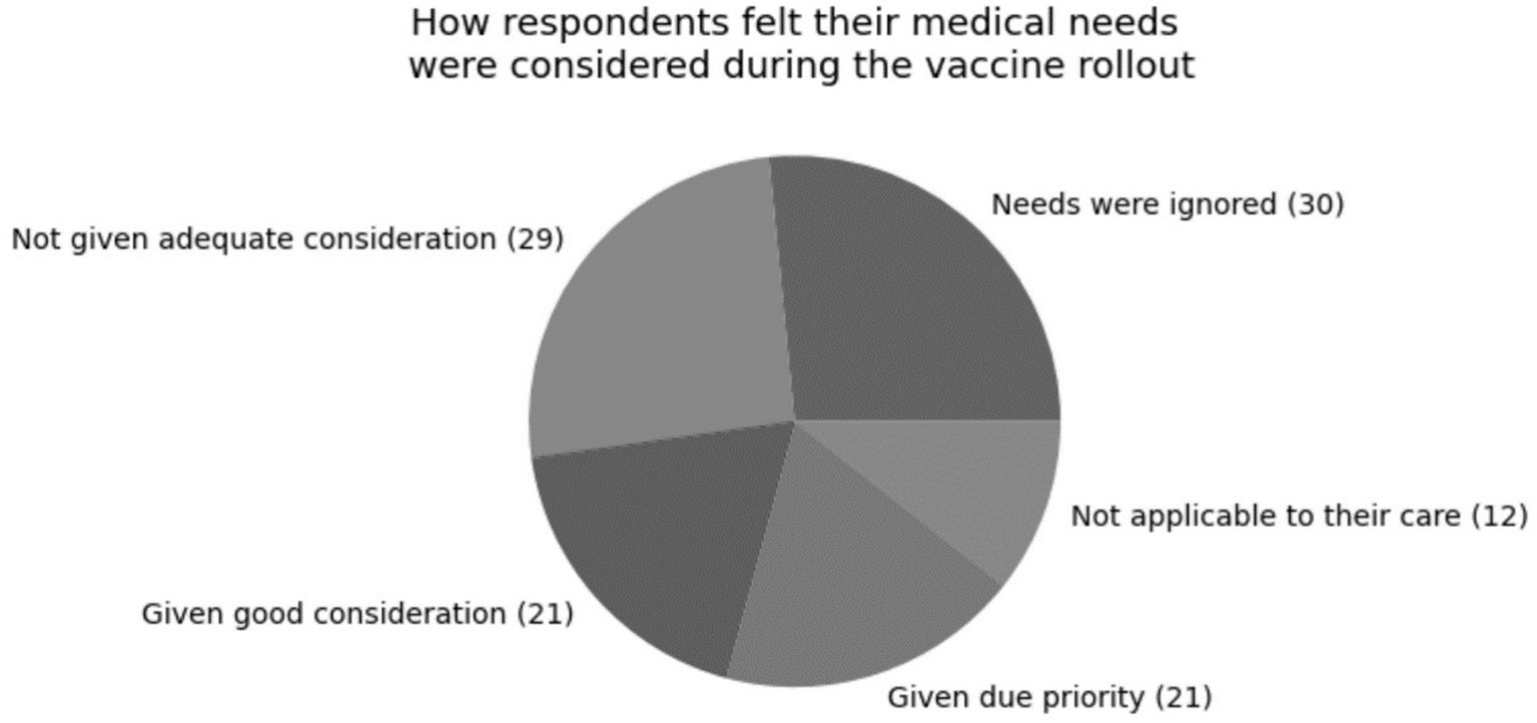
How respondents felt their medical needs were considered during the vaccine rollout. Of the 113 respondents to this question, 30 (27%) felt like they had been ignored, 29 (26%) felt they had not been given adequate consideration, 21 (19%) felt they had been given good consideration, 21 (19%) felt they had been given due priority, and the remainder did not feel this question was applicable to their care.

Of the 123 respondents to this question, 84 (68%) had received the vaccine. When asked if respondents felt their medical needs had been considered during the vaccine rollout, of the 113 respondents to this question, 30 (27%) felt like they had been ignored, 29 (26%) felt they had not been given adequate consideration, 21 (19%) felt they had been given good consideration, 21 (19%) felt they had been given due priority, and the remainder did not feel this question was applicable to their care. This is illustrated in Figure 2.

## Discussion

This study explores the perception of clinical care, health, and well-being by patients with osteogenesis imperfecta, and their carers, throughout the COVID-19 pandemic. As it stands, there are more than 626 million COVID-19 cases globally, and 6.5 million COVID-19 related deaths reported by WHO before October 25th, 2022 (12). The United Kingdom currently stands as the seventh highest in reported coronavirus cases (13), where the majority (85%) of respondents to these surveys were located.

This study has shown that appointment delays were very common in both 2020 and 2021 surveys. This could be due to the backlog of appointments across the pandemic and was associated with anxiety and frustration in one-sixth of the participants. These feelings were likely to be compounded as a large proportion of those with delayed appointments were not provided with new appointment dates.

Patients with rare diseases often rely on a combination of clinical interventions including medical therapies, medications, physiotherapy, and rehabilitation services. Discontinuation of healthcare services and new lockdown measures have greatly reduced the availability of these services with an adverse effect on mental health and well-being (1, 2). A recent study by EURORDIS has shown that 90% of patients have experienced a delay in their clinical care since the beginning of the pandemic (5), 6 in 10 have perceived these to be detrimental, and 3 in 10 have felt these delays are life-threatening. A further study showed that many patients with rare bone conditions have suffered from feelings of abandonment and fear (14).

In 89% of the initial survey participants, the lockdown resulted in their first experience of remote consultation. Only one-fifth of respondents rated the virtual consultation as “*not good*” or “*poor*”. A recent study showed that 90% of respondents who had a teleconsultation were happy with the experience and felt it was useful (5). With over half of respondents in the first survey transitioning to remote consultation, which increased to almost three-quarters in the second wave of the pandemic, it is reassuring to see the increase in remote consultations and to know the majority of participants are still continuing treatment with a healthcare practitioner. Remote consultations existed prior to COVID-19 and had the benefit of being able to overcome geographical barriers to consultations, especially for those with rare conditions, who often have to travel to major urban areas for specialist appointments (7). However, with the arrival of COVID-19, remote consultation moved from the primary domain of the assessment of stable patients to cover those with acute and rare conditions (15, 16). In the initial survey approximately half of participants said that they would like more remote consultations, increasing in the follow-up survey to 71% who would prefer remote consultations either for all or some appointments. Challenges faced in remote consultations include difficulty establishing rapport, limitations to examinations and difficulty with complex concepts (7), highlighted by one-fifth of participants who described challenges with lack of examinations and blood tests. Alongside these, there are also ethical and legal challenges to telehealth (1) and a requirement for patients to have digital access and expertise to join a remote consultation (17).

Those with rare conditions already experience higher levels of depression and anxiety than the general population, along with high levels of isolation and reduced interaction with family even prior to the need for shielding and social distancing (18, 19). Pre-existing mental health disorders have been worsened by the fear and uncertainty of the COVID-19 pandemic, and the need for shielding and social distancing has increased feelings of social isolation and loneliness (7, 20, 21). Individuals with OI may have a reduced quality of life when compared with the general population, in particular, those with a more severe degree of OI reporting a worse quality of life (22). Although a pre-pandemic survey in the current cohort of participants was not available to establish a baseline, both surveys revealed that a substantial proportion of participants were struggling with anxiety. In the primary survey, when asked how COVID-19 was impacting their mental health 13% mentioned lack of contact with friends and family, increasing in the second survey to 84% when asked to rate how seeing friends and family affected them on a scale of not at all to significantly. Anxiety due to the lockdown was identified in over 60% of respondents in both surveys. The other theme identified in both surveys was difficulty keeping physically active. Physical therapy is also an important aspect of the management of OI, for achieving maximal bone density but also optimizing psychological well-being (23). Since the beginning of the pandemic, two out of three patients with rare conditions struggled with depression and/or felt unable to overcome issues (24). Previous studies investigating the effects of mental health in patients with rare conditions have shown that 80% of patients have suffered during the pandemic, with those who are dependent affected more than those who live life independently (2). This is echoed in our study, which showed that the percentage of those struggling with mental health increased from 44% of those without carers to 76% for those with carers. Almost half of the respondents who required regular support or personal assistance at home had their hours either completely or partially reduced, which can create challenges at home with significant stress for the primary carers (25).

Whilst the UK Government did not specifically mention OI as a clinically extremely vulnerable group that should shield (26) some people with OI may be considered to be at a greater risk of more severe disease and pulmonary complications (27). Almost one-third of participants in this study received a public health letter advising they were at risk of severe illness secondary to COVID-19, but over half had to self-identify as being at risk, due to underlying health conditions. In other research, 60% of people with rare conditions stated they had difficulty obtaining the information they required on COVID-19 (24). A substantial proportion of people with OI changed their behavior around seeking medical help with a fifth not attending hospital for a suspected fracture and almost half changing their therapies or appointments to avoid attending hospital. This could have led to greater pain and discomfort due to a lack of appropriate immobilization and may have increased the potential for a poorer outcome long term. Additionally, over half of respondents to the first survey had rarely left their homes since the start of the pandemic in March 2020.

By the time of the April 2021 survey, the majority of participants had received their first vaccine. However, over half of respondents felt that their medical needs were not prioritized correctly with regard to the vaccination rollout. This is not an uncommon experience for those with rare conditions, as knowledge of their conditions is often limited (28). There is a need for countries to address the unmet need for investment in prevention of illness for those with rare diseases *via* clinical research and development activities (29). It is reassuring however that evidence to date shows that COVID-19 infection does not have a particularly adverse effect on those with OI (30). During the pandemic, the BBS provided online COVID-19 information to its members, with the majority of respondents finding this very helpful. Previous studies have shown that online support from patient organizations is welcomed amongst patients with rare diseases (3, 31) and in the case of some rare conditions are potentially the only source of information (32). With patient organizations being described as the major lifeline for many of the rare disease communities (1), it is unfortunate that many of these are currently working at reduced or no capacity due to decreased staffing, lack of fundraising, and diminished government subsidies (7).

Although one of the strengths of this study was the comparison of opinions across two consecutive years of the pandemic, unfortunately, we do not have a pre-pandemic survey in this cohort of participants to establish a baseline of regular clinical care, which is particularly important when trying to assess differences in appointment delays, mental health and therapy options. We also do not have a clear measure of the number of people who would have seen the survey or opted not to complete it. Given that the survey responses were collected anonymously, it is also theoretically possible that the same person completed a questionnaire more than once. The survey's questions also changed significantly from year to year, making direct comparison challenging. Respondents to the second survey also lived outside the UK and Ireland, unlike the first survey. As with many qualitative studies, there are numerous instances of missing data (1). Furthermore, this study did not look at the delay in diagnosis as the survey was only circulated to those with known OI. It would also be helpful to explore the impact of the pandemic on carers. Lastly, the majority of responses related to adults with OI, with only 11 respondents within the childhood age bracket and adequate comparison to children would have required a larger number of cases.

In summary, these surveys have revealed that the pandemic has had widespread effects on both the mental and physical health of those with OI. These effects, alongside appointment delays, have increased as the pandemic progresses. Encouragingly, the increasing preference for remote consultation as a direct consequence of COVID-19 may prove a viable long-lasting alternative to face-to-face appointments, especially for patients who previously traveled vast distances for specialist care, and may be a solution for the backlog of NHS appointments due to the ongoing pandemic (33). It would be beneficial to perform this survey at occasional but regular intervals to assess the ongoing impact of COVID-19, and to explore pandemic-related healthcare in a wider range of countries, to obtain a broader and more global perspective.

## Data availability statement

The original contributions presented in the study are included in the article/supplementary material, further inquiries can be directed to the corresponding author.

## Ethics statement

The studies involving human participants were reviewed and approved by all responses to the surveys were anonymous. The Brittle Bone Society has a Data Protection Policy, adheres to GDPR (General Data Protection Regulation) regulations, and is registered with the ICO (Information Commissioner's Office). Written informed consent to participate in this study was provided by the participants' legal guardian/next of kin.

## Author contributions

MH, DS, CK, and SA undertook the initial data analysis and wrote the initial draft. The BBS developed both questionnaires and distributed these to participants. All authors contributed to the revision of the manuscript and have read and approved the final report, take public responsibility, and accountability for the results.
